# Effect of 10.6 μm laser moxibustion on inflammation in diabetic peripheral neuropathy rats

**DOI:** 10.3389/fendo.2023.1203677

**Published:** 2023-08-01

**Authors:** Yue Yin, Lina Wang, Ling Zhao, Lin Lin, Xueyong Shen

**Affiliations:** ^1^ School of Acupuncture-Moxibustion and Tuina, Shanghai University of Traditional Chinese Medicine, Shanghai, China; ^2^ School of Nursing, Shanghai University of Traditional Chinese Medicine, Shanghai, China; ^3^ Laboratory of Systemic Physiology for Acupuncture and Meridian, Shanghai Research Center of Acupuncture and Meridian, Shanghai, China

**Keywords:** diabetes mellitus, diabetic peripheral neuropathy, laser moxibustion, inflammation, NF- kappa B

## Abstract

Diabetic peripheral neuropathy (DPN) is the main cause of disability in diabetes patients but the efficacy of available drugs is poor. Moxibustion is an adjunctive treatment for DPN that can reduce symptoms. The peak value of the far infrared wavelength of 10.6 μm laser moxibustion is close to the infrared radiation spectrum of traditional moxibustion. Its effect is similar to that of moxibustion and does not cause pain, infection or produce irritating smoke. Twenty-four male SD rats were divided into control (Con), DPN, laser moxibustion (LM), and pyrrolidine dithiocarbamate (PDTC) groups (n=6/group). The DPN, LM and PDTC group rats were intraperitoneally injected with 1% streptozotocin (STZ) to induce a model of DPN. LM group rats were irradiated with a laser at bilateral ST36 acupoints for 15 min, once every other day, for 14 days. PDTC group rats were intraperitoneally injected with PDTC once a day. Body weight, blood glucose, and paw withdrawal mechanical threshold (PWMT) were measured and laser speckle imaging (LSI) performed before and after modeling and at 1 and 2 weeks after intervention. Two weeks after intervention, changes in serum interleukin 1β (IL1β), interleukin 6 (IL6), tumor necrosis factor α (TNFα) and nerve growth factor (NGF) were analyzed, and the abundance of NF-κB and IκB-α proteins and levels of NF-κB and IκB-α mRNAs in the sciatic nerve were observed. The results showed that 10.6 μm laser moxibustion can relieve pain, improve microcirculation, and alleviate inflammation in DPN rats, possibly via the NF-κB inflammatory pathway.

## Introduction

Diabetic peripheral neuropathy (DPN) is one of the most common chronic complications of diabetes mellitus (DM). According to recent estimates, the global prevalence of diabetes among individuals aged 20-79 was reported to be 536.6 million in 2021, with projections indicating an alarming rise to 783.2 million by 2045 (1). Approximately 50% of individuals diagnosed with diabetes are expected to experience DPN at some point during their lives (2). This condition not only profoundly impacts the quality of life and prognosis of affected patients but also exerts a considerable financial burden on healthcare systems (3). For instance, in England alone, the annual cost associated with the treatment of ulcers and amputations in individuals with diabetes was estimated to range between EUR 837 million and EUR 962 million (4). Similarly, in Brazil, the direct medical expenses attributed to diabetes-related foot complications, such as foot ulcers and amputations, are estimated to amount to $361 million annually (5).DPN mainly refers to symptoms related to peripheral nerve dysfunction in DM patients after other reasons have been excluded. These symptoms usually manifest as symmetrical numbness, pain and sensory abnormalities in the distal limbs, which first appear in the lower limbs, and in serious cases, skin ulceration and erosion can be seen, which is difficult to treat and can lead to amputation (6, 7). The pathogenesis of DPN is unclear. It is believed to be related to hyperglycemia, dyslipidemia, inflammatory reactions, oxidative stress and other factors (8, 9). Current treatments are mainly based on controlling the primary disease by regulating blood glucose. Supplementation with symptomatic drugs can only temporarily relieve symptoms and can have side effects (10, 11).

Acupuncture and moxibustion are widely practiced and their use is gradually increasing. Acupuncture and moxibustion can prevent and control the development of DPN (12). Moxibustion, a traditional Chinese medicine therapy, is used to stimulate acupoints through the heat generated by burning moxa. However, the burning of moxa can generate toxic smoke and burn the skin of patients, and in DPN patients there is an increased risk of infection (13). Laser moxibustion is the combination of traditional moxibustion and modern low-level laser therapy. It uses a low-intensity laser to irradiate acupoints, which produces thermal effects, and will not cause local pain or infection, or produce irritating smoke. We previously showed that 10.6 μm laser moxibustion can produce 10.6 μm wavelength far-infrared light, which is close to the infrared radiation spectrum of traditional moxibustion and human acupuncture points, and the three peaks are about 10 μm (14). Therefore, 10.6 μm laser moxibustion can have a lasting thermal effect (15), can affect deep tissue (16) and resonate with acupoints to produce therapeutic effects. The primary objective of our study is to offer researchers and individuals affected by DPN a treatment method that is safer, more suitable, and highly effective.

Inflammation is a main feature of DPN, involving a large number of inflammatory factors. Nuclear factor kappa B (NF-κB) participates in the regulation of most cytokines. NF-κB is inactive when bound to its inhibitor protein, NF-kappa-B inhibitor alpha (IκB-α). When NF-κB is activated, it can regulate the transcription of inflammatory factor genes, leading to the release of inflammatory factors, such as IL1β, IL6 and TNFα downstream of NF-κB, thereby initiating the inflammatory reaction (17). Therefore, the NF-κB signaling pathway can be targeted to improve the inflammatory response of DPN. In addition, to further validate the involvement of NF-κB in the inflammatory response associated with DPN, we have employed the well-established NF-κB-specific inhibitor PDTC (18–20). PDTC acts by inhibiting the degradation of IκB-α, thereby preventing the translocation of NF-κB from the cytoplasm to the nucleus, and effectively blocking the NF-κB signaling pathway (18, 19). Therefore, by utilizing the PDTC group as a control observation, we further explore whether 10.6μm laser moxibustion can have a therapeutic effect similar to PDTC in inhibiting inflammatory reactions. We previously confirmed that 10.6 μm laser moxibustion can affect inflammatory reactions (21, 22), but the exploration of inflammatory factors is preliminary, lacking an investigation into the influence on the NF-κB inflammatory pathway. This study aims to explore whether 10.6 μm laser moxibustion can inhibit inflammatory reactions and alleviate peripheral neuropathy in DM rats by regulating the NF-κB pathway, so as to provide a theoretical basis for laser moxibustion treatment of DPN.

## Methods

### Animals

Male Sprague-Dawley (SD) rats (weighing 200 ± 20 g; SCXK2018-0006; Xipuer-Bikey Co., Ltd., Shanghai, China) were housed in controlled conditions (20–24°C, relative humidity of 40–60%, and 12/12 h light-dark cycle) with food and water freely available. All experiments were approved by the Shanghai University of Traditional Chinese Medicine Animal Welfare and Ethics Committee (PZSHUTCM211018006) and conformed to the standards of the International Council for Laboratory Animal Science.

### DPN model and animal groups

Rats were randomly divided into four groups: control (Con), DPN, laser moxibustion (LM), and pyrrolidine dithiocarbamate (PDTC) groups. The rats in the DPN, LM and PDTC groups were fasted for 12 hours and then injected intraperitoneally with 60 mg/kg streptozotocin (STZ) (Sigma, USA) dissolved in 0.1 M sodium citrate buffer (23, 24). Seven days after STZ injection, successful DPN model induction was confirmed by a tail blood glucose level ≥ 16.7 mmol/L and the paw withdrawal mechanical threshold (PWMT) being decreased by ≥ 15% (25). Con group rats were injected intraperitoneally with the same volume of sodium citrate buffer.

Body weight and PWMT were measured before and after modeling, on the 15th and 21st day of the experiment. Blood glucose and laser speckle imaging (LSI) were measured after modeling on the 15th day and 21st day of the experiment. IL6, TNFα, IL1β, NGF, NF-κB and IκB-α proteins, and NF-κB and IκB-α mRNAs were measured 2 weeks after intervention. The schedule of the experimental scheme is shown in **Supplementary Figure 1A**.

### Animal treatment

The LM group underwent fixation using custom-made black and light-proof cloth bags to cover the head and upper limbs of the rats, while a 10.6 μm laser device (SX10-C1; Wonderful-Opto-Electrics Tech Co, Ltd., Shanghai, China) was applied bilaterally at the ST36 acupoints (between the anterior tibialis and the extensor digitorum longus muscles) every other day for a duration of 14 days, starting from the second day after modeling. See **Table 1** for the laser parameters and **Supplementary Figures 1B, C** for the experimental procedure. Rats in the PDTC group were subjected to the same grasping and fixation conditions as the LM group and received 100 mg/kg PDTC (intraperitoneal injection) every day for 14 days (26). The Con group rats were neither modeled nor subjected to any treatment. The DPN group rats were successfully modeled but did not receive any further treatment. During the experiment, the Con and DPN groups were subjected to the same grasping and fixation conditions as the LM groups, except without the laser intervention.

**Table 1:** The parameters of laser moxibustion.

10.6 μm laser device	Parameters
Wavelength	10.6 μm
Number and type of emitters	CO_2_ laser
Pulse mode	Continuous pulse
Radiant power	80 mW
Spot diameter	2 mm
Treatment duration	15 min/acupoint point (30 min/each time)
Power density	2.55 W/cm^2^
Energy density	2295.00 J/cm^2^
Each treatment dose	144.12 J
Treatment frequency	Once every 2 days (total seven applications)

### Measurement of blood glucose

Non-fasting blood glucose levels of all rats were measured between 10:00-11:00 am. Tail vein blood samples were taken and blood glucose values recorded using a meter (27) (HGM-114, Omron, Suzhou Erda Medical Equipment Co., Ltd., China).

### Paw withdrawal mechanical threshold testing

Prior to testing, rats were acclimatized to the experimental environment for 30 min. An Electronic VonFry Aesthesiometer (IITC Life Science) was used to stimulate the central sole area of the feet. The force was evenly increased until the rats had a claw withdrawal reaction; this force is the PWMT (21). The measurement was repeated three times for each rat with an interval of 10 minutes and the average value calculated. Finally, the PWMT of each rat is the average stimulation intensity for bilateral hind feet.

### Laser speckle imaging

Before the test, the rats were fixed and acclimatized to the environment for 5 minutes. A FLPI-2 Full Field Laser Perfusion Imager (LSI FLPI-2) from Moor Instruments Ltd, UK was used. The infrared laser points were focused on the dorsal surface of the rats’ hind feet, and the blood perfusion volume of the rats’ skin was measured. Moor FLPI-2 measurement software V2.0 was used to record the data, and V5.0 software was used to process the laser speckle images and data (28). The final values are the average of both sides.

### Enzyme-linked immunosorbent assay

All rats were sampled after 2 weeks of intervention. After fasting for 12 hours overnight and weighing, all rats were anesthetized with 40 mg/kg pentobarbital sodium. After the rats entered deep anesthesia, blood from the abdominal aorta was collected and centrifuged (at 4°C, 3000 r/min, 15 min). The supernatant was stored at -80°C. Rat ELISA kits (ZCIBIO, China) were used to analyze IL6, IL1β, TNFα and NGF according to the manufacturer’s instructions.

### Western blotting

After taking blood from the abdominal aorta, the rat sciatic nerve was quickly dissected under sterile conditions. Samples were homogenized in lysis buffer and supernatant collected after centrifugation at 12000 rpm/min, 10min at 4°C. Protein concentration in the supernatant was determined by the bicinchoninic acid protein assays. Samples were subjected to 10% sodium dodecyl sulfatepolyacrylamide gel electrophoresis (SDS-PAGE) and then transferred to a polyvinylidene fluoride (PVDF) membrane. Following overnight blocking with 5% bovine serum albumin (BSA), blots were incubated at 4°C overnight with the following primary antibodies: anti-p65 subunit of NF-κB antibody (1:1000; Abcam, USA) and anti-IκB-α antibody (1:1000; Abcam), and then with a horseradish peroxidase (HRP)-conjugated secondary antibody at room temperature (RT) for 2 h (29). Enhanced chemiluminescence (ECL) was used to visualize the blots, and alphaEaseFC (Alpha Innotech, USA) was used to analyze the density of specific bands.

### Quantitative real time-PCR

Total mRNA was isolated from sciatic nerves. cDNA was synthesized using a Servicebio^®^RT First Strand cDNA Synthesis Kit (ZCIBIO, China) according to the manufacturer’s instructions. 2×SYBR Green qPCR Master Mix (ZCIBIO, China) was used for real-time PCR (30). The NFKB-P65 primers were: sense strand, 5′-CAGATACCACTAAGACGCACCC-3′ and antisense strand, 5′-CTCCAGGTCTCGCTTCTTCACA-3′. The IKBα primers were sense strand, 5′-CCCAAGTACCCGGATACAGCA-3′ and antisense strand, 5′-GTCATCGTAGGGCAACTCATCTT-3′. The relative abundance of specific mRNAs was determined by the 2-ΔΔCT method, using GAPDH as the internal standard. The GAPDH primers were: sense strand, 5′-CTGGAGAAACCTGCCAAGTATG-3′ and antisense strand, 5′-GGTGGAAGAATGGGAGTTGCT-3′.

### Statistical analysis

All data are expressed as the mean ± SEM. SPSS 29.0 (IBM SPSS Statistics, New York, USA) statistical analysis software was used for statistical analysis, and GraphPad Prism9.0 software (La Jolla, CA, United States) was used to draw graphs. *P* < 0.05 was taken to indicate statistical significance. Blood glucose, body weight, PWMT and LSI were evaluated by two-way ANOVA with repeated measures, followed by a *post-hoc* least significant difference test. ELISA, western blotting and qPCR results were analyzed by one-way ANOVA to evaluate differences among multiple groups, followed by a *post-hoc* least significant difference test.

## Results

### Comparison of weight and blood glucose levels among groups

Con group rats had normal food intake, water consumption and urine production, good activity, white, smooth and shiny fur, and continued to gain weight. After establishment of the model, the rats in the other three groups significantly increased their intake of food and drinking water and produced more urine compared with the Con group. Furthermore, they appeared thin, their fur was yellow, slippery, disheveled and lacked glossiness, and the cage litter was dirty and smelly.

Following the modeling, body weights of the DPN group remained consistently lower than that of the Con group (*P* < 0.001). On the 21st day, the body weight of the PDTC group was higher than that of the DPN group (*P* < 0.01) and the LM group (*P* < 0.01) (**Supplementary Figure 1D**). Moreover, after modeling, the blood glucose levels in the DPN group remained significantly higher than those in the Con group (*P* < 0.001). At each time point, there was no significant difference between the two intervention groups and the DPN group (*P* > 0.05). (**Supplementary Figure 1E**).

### Comparison of paw withdrawal mechanical threshold among groups

**Figure 1:**
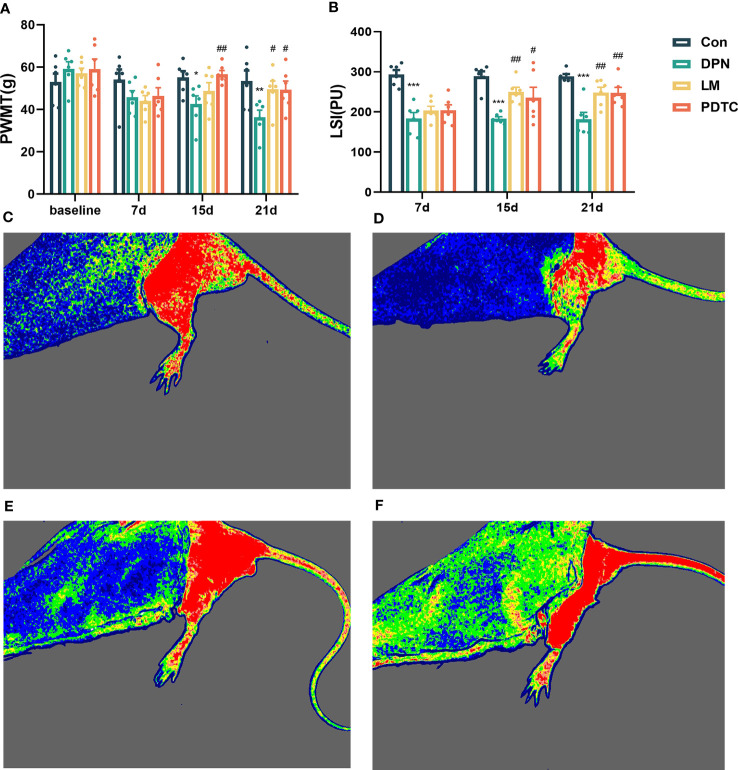
**(A)** Comparison of mechanical pain threshold among groups (n = 6, the mean ± SE). Two-way ANOVA followed by LSD was used. ^*^ *P* < 0.05, ^**^ *P* < 0.01, compared with the Con group; ^#^ *P* < 0.05, ^##^ *P* < 0.01, compared with the DPN group. **(B)** Comparison of skin blood perfusion volume among groups (n = 6, the mean ± SE). Two-way ANOVA followed by LSD was used. ^***^ *P* < 0.001, compared with the Con group; ^#^ *P* < 0.05, ^##^ *P* < 0.01, compared with the DPN group. Comparison of laser speckle imaging among groups: **(C)** Con; **(D)** DPN; **(E)** LM; **(F)** PDTC.

On the 7th day, the PWMT decreased in the three modeling groups. However, there was no statistically difference in the PWMT among the four groups (*P* > 0.05). By the 15th day, the PWMT of the DPN group was significantly lower than that of the Con group (*P* < 0.05). The PWMT of the PDTC group was significantly higher than that of the DPN group (*P* < 0.01). On the 21st day, the PWMT of the DPN group continued to decrease compared to the Con group (*P* < 0.01). In contrast, both the LM and PDTC groups exhibited an increase in PWMT compared to the DPN group (*P* < 0.05) (**Figure 1A**).

### Comparison of laser speckle imaging among groups

After modeling, the skin blood perfusion volume of rats in DPN group remained significantly lower than that in the Con group (*P* < 0.001). By the 15th day, the skin blood perfusion volume of both the LM group (*P* < 0.01) and PDTC group (*P* < 0.05) significantly increased compared to the DPN group. On the 21st day, the skin blood perfusion volume of both the LM and PDTC groups also significantly increased compared to the DPN group (*P* < 0.01). (**Figures 1B–F**).

### Comparison of serum nerve growth factor among groups

**Figure 2:**
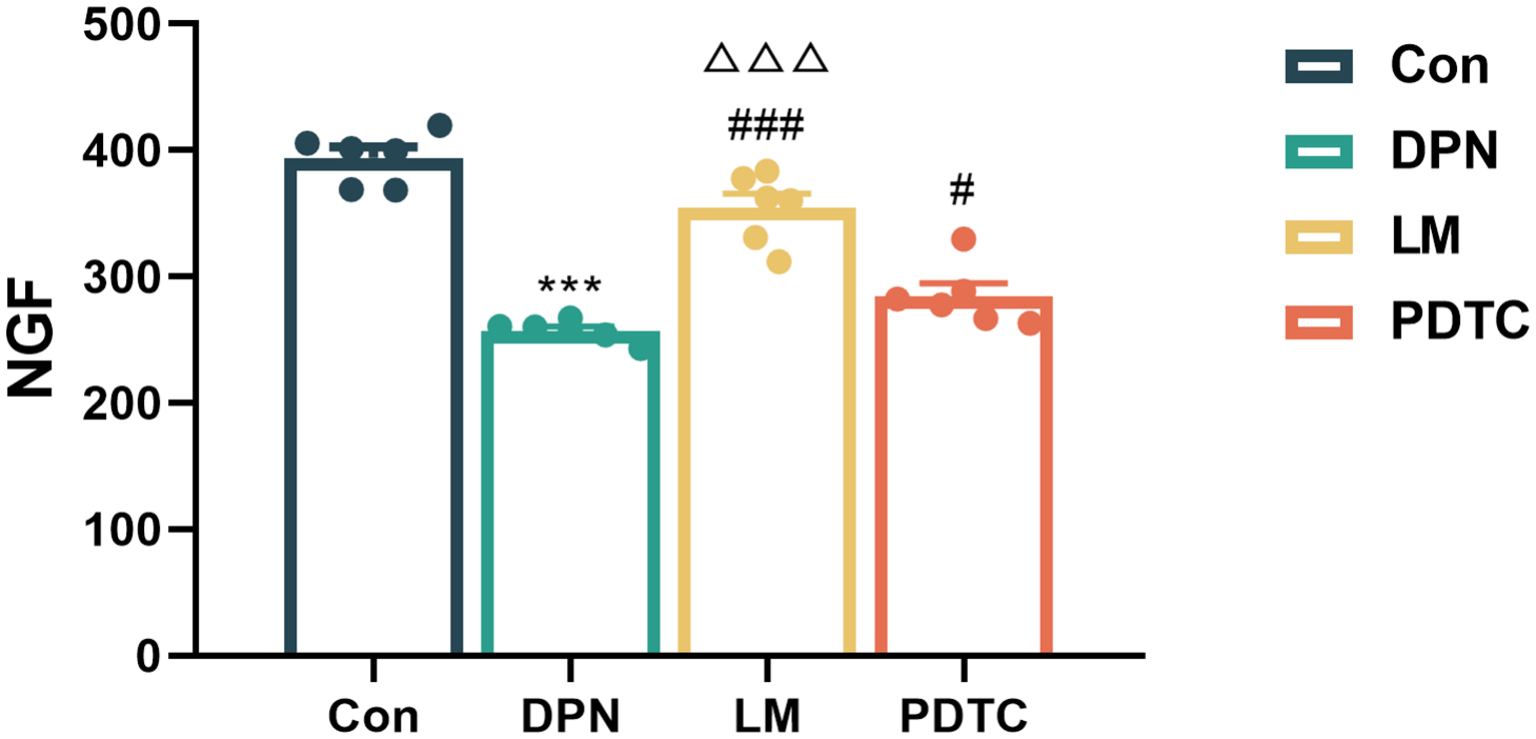
Comparison of serum NGF among groups (n = 6, the mean ± SE). One-way ANOVA followed by LSD was used. ^***^ *P* < 0.001, compared with the Con group; ^#^ *P* < 0.05, ^###^ *P* < 0.001, compared with the DPN group; ^△△△^ *P* < 0.001, compared with the PDTC group.

Upon intervention, serum NGF levels in the DPN group were significantly lower compared to the Con group (*P* < 0.001). Conversely, the NGF content in both the LM group (*P* < 0.001) and PDTC (*P* < 0.05) group showed a significnat increase when compared to the DPN group. Moreover, the NGF content in the LM group was found to be higher compared to the PDTC group(*P* < 0.001). (**Figure 2**).

### Comparison of serum inflammatory factors among groups

**Figure 3:**
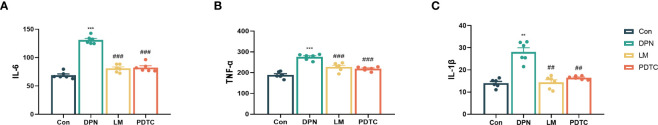
Comparison of serum inflammatory factors among groups. **(A)** IL6; **(B)** TNFα; **(C)** IL1β. (n = 6, the mean ± SE). One-way ANOVA followed by LSD was used. ^**^ *P* < 0.01, ^***^ *P* < 0.001, compared with the Con group; ^##^ *P* < 0.01,^###^ *P* < 0.001, compared with the DPN group.

After intervention, serum IL6 in the DPN group was significantly higher than that in the Con group (*P* < 0.001). However, both the LM and PDTC groups exhibited significantly lower IL6 levels when compared to the DPN group (*P* < 0.001) (**Figure 3A**). Similarly, Serum TNFα in the DPN group was significantly higher than that in Con group (*P* < 0.001). However, both the LM and PDTC groups showed lower TNFα levels when compared to the DPN group (*P* < 0.001) (**Figure 3B**). IL1β in the DPN group was significantly higher than that in the Con group (*P* < 0.01). Compared with DPN group, IL1β was significantly decreased in the LM and PDTC groups (*P* < 0.01) (**Figure 3C**).

### Comparison of NF-κB and IκB-α among groups

**Figure 4:**
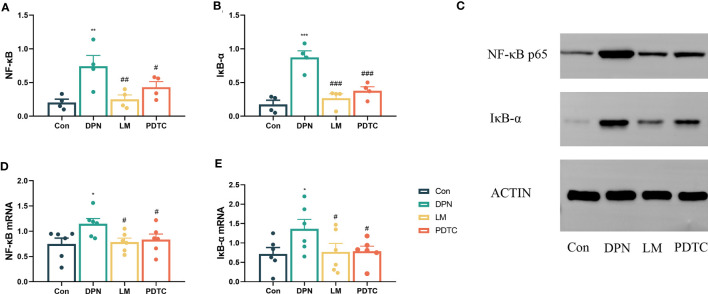
Comparison of NF-κB **(A)** and IκB-α **(B)** protein levels in the sciatic nerve among groups. (n = 4, the mean ± SE). One-way ANOVA followed by LSD was used. ^**^ *P* < 0.01, ^***^ *P* < 0.001, compared with the Con group; ^#^ *P* < 0.05, ^##^ *P* < 0.01, ^###^ *P* < 0.001, compared with the DPN group. **(C)** Western blot measurement of sciatic nerve proteins. Comparison of NF-κB **(D)** and IκB-α **(E)** mRNAs in the sciatic nerve among groups. (n = 6, the mean ± SE). One-way ANOVA followed by LSD was used. ^*^ *P* < 0.05, compared with the Con group; ^#^ *P* < 0.05, compared with the DPN group.

After intervention, the abundance of NF-κB protein in the sciatic nerve of the DPN group was significantly higher than that in the Con group *(P* < 0.01). Compared with the DPN group, NF-κB abundance was significantly decreased in the LM group (*P* < 0.01) and the PDTC group (*P* < 0.05) (**Figures 4A, C**).

Moreover, the abundance of IκB-α in the sciatic nerve of the DPN group was significantly higher than that in the Con group (*P* < 0.001). However, both the LM and PDTC groups exhibited a significant decrease in IκB-α abundance when compared to the DPN group (*P* < 0.001) (**Figures 4B, C**).

### Comparison of NF-κB and IκB-α mRNAs among groups

Subsequent to the intervention, the level of NF-κB and IκB-α mRNA in the sciatic nerve of the DPN group was increased compared to the Con group (*P* < 0.05). Compared with the DPN group, the level of NF-κB and IκB-α mRNA in the sciatic nerve of LM and PDTC groups was significantly decreased (*P* < 0.05) (**Figures 4D, E**).

## Discussion

Acupuncture and moxibustion is a simple and effective therapy that can improve the clinical symptoms and quality of life of patients with DPN (31–33). In addition, animal experiments have confirmed that acupuncture and moxibustion can potentiate nerve damage repair in diabetes (34–36). Acupuncture and moxibustion have been increasingly applied to the treatment of DPN (37). Compared with traditional moxibustion, the use of lasers has significant advantages for treating diabetes and its complications (38, 39); however, its therapeutic mechanism has not been determined.

Continuously high levels of glucose damage nerve cells and are strongly associated with DPN. Although the blood glucose of DPN model rats increased, glucose could not be properly absorbed and utilized. Protein and fat became the main energy sources, leading to significant weight loss. The blood glucose in the three model groups increased significantly, their weight decreased, and clinical manifestations of DM, including polydipsia, greater food intake, and polyuria appeared. The blood glucose level in each intervention group did not decrease significantly during treatment. There was no significant improvement in body weight in any intervention group compared with the DPN group after 1 week of intervention. After 2 weeks of intervention, weight was significantly increased in the PDTC group only. These results showed that after 2 weeks of 10.6 μm laser moxibustion intervention, there was no significant change in body weight or blood glucose in STZ-induced DPN rats.

After model establishment, the PWMT and body weight decreased significantly and blood glucose increased, indicating that the DPN model was successfully established. The PWMT of the DPN group continued to decline with time. After 1 week of intervention, the PWMT of the PDTC group was significantly increased, and the PWMT of rats in the LM group was also increased, but not significantly. After 2 weeks of intervention, the PWMT of the LM and PDTC groups was significantly higher than that of the DPN group, indicating that both 10.6 μm laser moxibustion and PDTC can increase the PWMT of STZ-induced DPN rats, but that 10.6 μm laser moxibustion may take longer to improve the PWMT than PDTC.

DPN is one of the most common microvascular complications of diabetes. LSI can measure local skin microcirculation (40). The change of blood perfusion volume can reflect the state of microcirculation. When a microvessel is abnormal, it can cause nerve dysfunction. The blood flow in the dorsal skin of the feet of the DPN, LM and PDTC groups was significantly lower than that in the Con group after the model was established, indicating that blood flow in the extremities of the DPN animal model changed greatly, and that there was a vascular microcirculation disorder. After the intervention, the blood perfusion volume of the dorsal skin of both feet was significantly higher in LM and PDTC groups than in the DPN group. 10.6 μm laser moxibustion and PDTC significantly improved the blood perfusion volume of DPN rats.

NFG promotes the growth of neurites and the proliferation of vascular cells. It plays an important role in the development and maturation of the nervous system, and in repair after neuronal injury (41). In animal studies, exogenous mouse NGF can affect the occurrence and development of mechanical hyperalgesia (42) and can alleviate neuropathic pain in a model of diabetes (43). In this experiment, serum NGF in DPN group rats decreased significantly, indicating that there may be nerve damage. After 2 weeks of intervention, serum NGF in LM and PDTC group rats increased significantly. The results showed that 10.6 μm laser moxibustion treatment can increase the level of serum NGF in DPN rats and improve nerve damage.

Inflammation is closely related to diabetes and its complications. IL6, TNFα and IL1β are main proinflammatory factors and play important roles in inflammation (44–47). In this experiment, we detected an inflammatory cascade driven by IL6, TNFα and IL1β in the serum of DPN group rats, which confirmed that inflammation was involved in the occurrence of DPN. After 2 weeks of intervention, the serum levels of IL6, TNFα and IL1β in the two intervention groups were significantly lower than those in the DPN group. This is consistent with the results of 10.6 μm laser moxibustion on IL6, TNFα and IL1β in knee osteoarthritis animal models (21, 22).

PDTC can inhibit NF-κB signal transduction to produce an anti-inflammatory effect and reduces the expression of pro-inflammatory cytokines (48). Therefore, we used PDTC as an inhibitor control group to confirm that the effect of 10.6 μm laser moxibustion on DPN rats occurs through NF-κB signaling. After intervention, the abundance of NF-κB and IκB-α in the sciatic nerve was significantly higher in the DPN group compared with the Con group, and significantly lower in the LM and PDTC groups than in the DPN group. In the DPN group, NF-κB and IκB-α mRNAs were significantly higher than those in the Con group. After 10.6 μm laser moxibustion and PDTC intervention, NF-κB and IκB-α mRNAs were significantly decreased. These results therefore indicate that 10.6 μm laser moxibustion can have a similar effcet to PDTC. It hinhibits inflammation in DPN rats induced by STZ and may affect nerve repair, possibly through the NF-κB inflammatory pathway.

This study primarily serves as an initial exploration of the mechanism underlying the effects of 10.6μm laser moxibustion on DPN. A notable limitation of this study is the selection of a single acupoint, whereas clinical treatments typically involve the combination of multiple acupoints. Future investigations should delve into the effectiveness of multi-acupoint interventions. Additionally, further research should integrate microscopic and molecular analyses of nerve conduction to elucidate the precise mechanism of action of 10.6μm laser moxibustion. Moreover, it is essential to incorporate human clinical studies to observe and assess its efficacy in a clinical setting.

## Conclusions

10.6 μm laser moxibustion can relieve pain, improve microcirculation, stimulate nerve repair and alleviate inflammation in DPN rats. Its effect on inflammation may be achieved by regulating the NF-κB inflammatory pathway.

## Data availability statement

The raw data supporting the conclusions of this article will be made available by the authors, without undue reservation.

## Ethics statement

All experiments were approved by the Shanghai University of Traditional Chinese Medicine Animal Welfare and Ethics Committee (PZSHUTCM211018006) and conformed to the standards of the International Council for Laboratory Animal Science.

## Author contributions

LL, LW, LZ, XS designed the experiment. YY, LL conducted the experiments. YY, XS analyzed the data. YY wrote the manuscript. All authors contributed to the article and approved the submitted version.

## Supplementary material

Supplementary Figure 1 Experimental procedure and general situation of DPN rats. **(A)** The schedule of the experimental protocol. The rat, syringe and centrifuge tube pictures are all from https://scidraw.io/. **(B)** 10.6 μm laser device. **(C)** Laser irradiation treatment. Comparisons of body weight **(D)** and blood glucose **(E)** among groups. (n=6, the mean ± SE). Two-way ANOVA followed by LSD was used. ^***^ *P* < 0.001, compared with the Con group; ^##^ *P* < 0.01, compared with the DPN group; ^△△^ *P* < 0.01, compared with the PDTC group.
