# Supplementary figures and images for: Effect of 10.6 μm laser moxibustion on inflammation in diabetic peripheral neuropathy rats
Source: Front Endocrinol (Lausanne). 2023 Aug 1;14:1203677. doi: 10.3389/fendo.2023.1203677 (PMC10427917; doi:10.3389/fendo.2023.1203677)

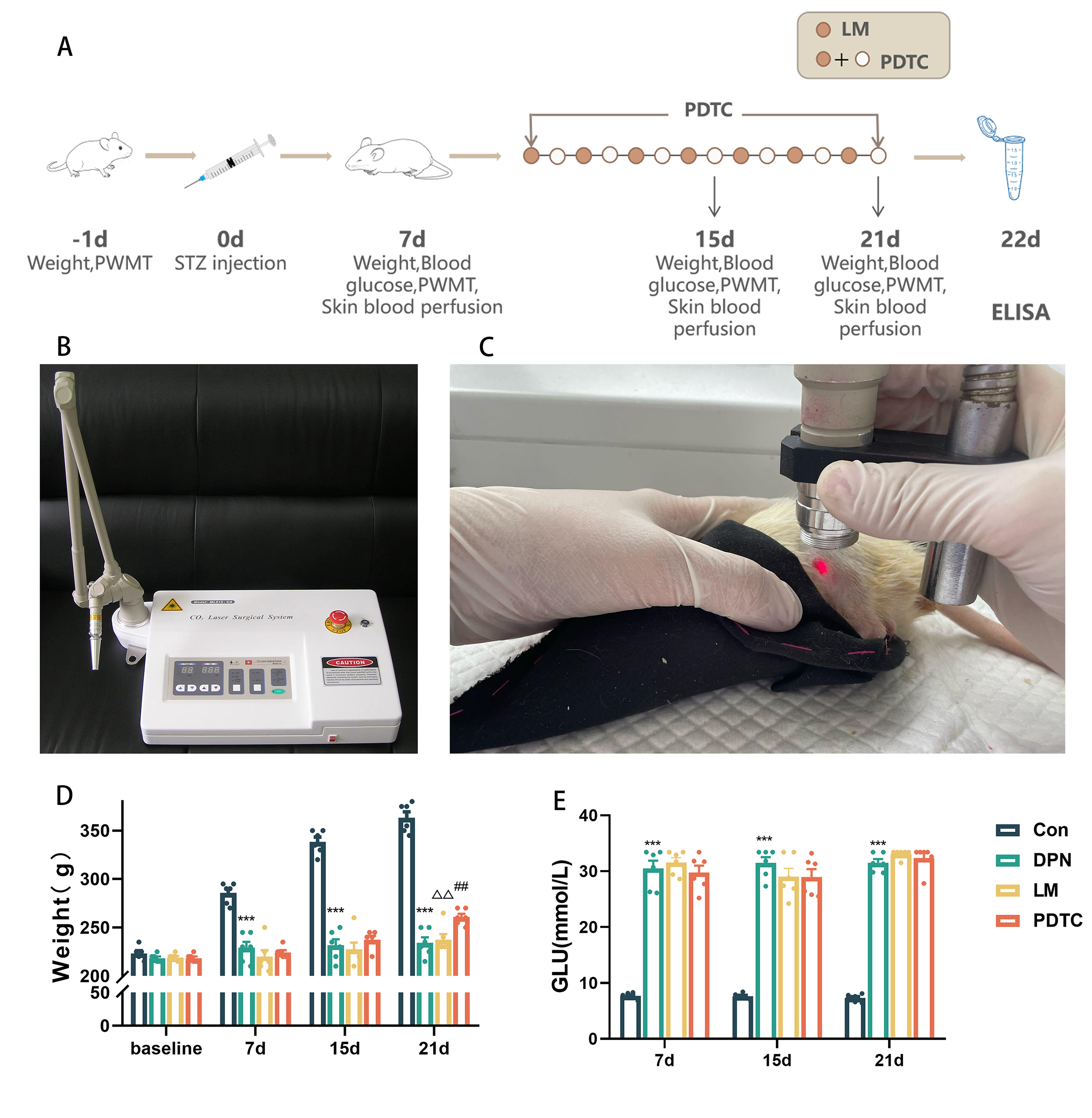

Supplement: Supplementary Figure 1 — Experimental procedure and general situation of DPN rats. (A) The schedule of the experimental protocol. The rat, syringe and centrifuge tube pictures are all from https://scidraw.io/. (B) 10.6 μm laser device. (C) Laser irradiation treatment. Comparisons of body weight (D) and blood glucose (E) among groups. (n=6, the mean ± SE). Two-way ANOVA followed by LSD was used. *** P < 0.001, compared with the Con group; ## P < 0.01, compared with the DPN group; △△ P < 0.01, compared with the PDTC group. [file Image_1.tif]
